# New Biomarkers of Hypertension and Related Vascular Disorders

**DOI:** 10.1155/2020/5075242

**Published:** 2020-07-20

**Authors:** Hao Peng, Chao Li, Changwei Li

**Affiliations:** ^1^Department of Epidemiology, School of Public Health, Medical College of Soochow University, Suzhou, China; ^2^Jiangsu Key Laboratory of Preventive and Translational Medicine for Geriatric Diseases, Suzhou, China; ^3^Department of Medicine, Queen Mary Hospital & State Key Laboratory of Pharmaceutical Biotechnology, University of Hong Kong, Hong Kong; ^4^Department of Epidemiology, Tulane University School of Public Health and Tropical Medicine, New Orleans, LA 70112, USA

As a very common risk factor of cardiovascular disease (CVD) and premature death, hypertension is preventable, and extensively controlling blood pressure to targets set forth by the current guideline is key to prevent and manage the debilitating disorder and CVD. In recent decades, the advancement of multiomics studies including genomics, epigenomics, transcriptomics, proteomics, metabolomics, and gut microbiome has uncovered many potential biomarkers for hypertension and its related cardiovascular complications including myocardial infarction, heart failure, and stroke [1–3]. Due to limited budget, most omics studies adopted a case-control study design which precludes causal inference. A better understanding of the causality and molecular mechanisms underlying the associations between the reported biomarkers and hypertension will undoubtedly promote clinical translation of the findings from omics studies. Therefore, follow-up studies targeting at those reported biomarkers are urgently needed. The aim of this special issue is to present recent progress in these exciting fields. A brief summary of all accepted papers is provided as follows.

The paper written by Dr. X. Peng et al. reported a longitudinal association between dynamic fasting glucose and risk of incident stroke in 12,321 Chinese adults. They found that a higher variance in addition to a higher level of fasting glucose predicted a higher risk of incident stroke, independent of lifestyle behaviors and metabolic factors. These results suggest that high variability of fasting glucose may be a risk factor for stroke. The homeostasis of glucose metabolism should be maintained for community members, diabetic patients in particular.

In the paper written by Dr. M.-C. Chen et al., the authors examined whether serum angiopoietin-like protein 3 (ANGPTL3) levels at baseline could predict adverse cardiovascular events in 90 patients with coronary artery disease (CAD). After a median follow-up of 54 months, 33 patients developed major adverse cardiovascular events, and these patients had higher ANGPTL3 levels at baseline compared with those who were free of adverse cardiovascular events during the follow-up. Cox regression models revealed that a higher baseline level of ANGPTL3 was significantly associated with an increased risk of developing adverse cardiovascular events for CAD patients. ANGPTL3 may be a predictor for major adverse cardiovascular events in CAD patients, but the efficacy and effectiveness of screening ANGPTL3 for CAD patients warrant further evaluation.

Leveraging data from 285 ischemic stroke patients receiving intravenous r-tPA thrombolysis within 4.5 hours of stroke onset, Dr. Y.-L. Liu and colleagues found that higher neutrophil-to-lymphocyte ratio at admission predicted risks of hemorrhagic transformation after r-tPA thrombolysis in acute ischemic stroke patients. Neutrophil-to-lymphocyte ratio, a parameter that can be easily estimated from blood cell count analysis, could be a useful marker for predicting hemorrhagic transformation in acute ischemic stroke patients after intravenous r-tPA thrombolysis.

The paper written by Dr. J. Zhang and colleagues reported a modification effect of platelet count on the association between homocysteine and blood pressure in more than 30,000 hypertensive patients. The association between homocysteine and blood pressure was much stronger in participants having low platelet count, compared with those having high platelet count. These results indicate that platelet count may be useful in the identification of individuals who are major beneficiaries of reducing-homocysteine treatments, but the clinical application still have a long way to go.

Dr. H. Xie et al. conducted a nested case-control study to examine the association between plasma urotensin II and the risks of incident hypertension by exploring 723 participants who developed hypertension and 1,096 participants who remained free of hypertension after about 5 years of follow-up. In contrast to prior case-control studies, they did not observe a significant association between urotensin II and hypertension. The causal effect of urotensin II on hypertension is still unspecified.

Dr. X. Jia et al. conducted an animal study to test the effect of resveratrol on pregnant hypertension reduction using mice models of high-salt diet-induced hypertension. They found that resveratrol supplementation could decrease blood pressure, serum urea, serum creatinine, and urinary protein in hypertensive pregnant mice. Resveratrol could be a promising candidate of an effective blood pressure lowering agent for pregnancy-induced hypertension, but further evidence is needed from clinical trials in humans.

Learning the new biomarkers of hypertension may help clinicians and researchers to understand the underlying mechanisms and provide additional motivation for undertaking the difficult challenge to reduce hypertension and its related cardiovascular disorders.
